# Unveiling the rumen-microbiome-brain circuit: a unique dimension of gut-brain axis in ruminants

**DOI:** 10.1186/s40104-025-01289-4

**Published:** 2025-11-27

**Authors:** Himani Joshi, Brandon Bernard, Caleb Lemley, Zhen Wang, Peixin Fan

**Affiliations:** 1 Department of Animal and Dairy Sciences, Mississippi State University, Mississippi State, MS 39762 USA; 2 Department of Biochemistry, Nutrition & Health Promotion, Mississippi State University, Mississippi State, MS 39762 USA; 3 Kimberly and Eric J. Waldman Department of Dermatology, Icahn School of Medicine at Mount Sinai, New York, NY 10028 USA; 4 Department of Immunology and Immunotherapy, Icahn School of Medicine at Mount Sinai, New York, NY 10028 USA; 5 Friedman Brain Institute, Icahn School of Medicine at Mount Sinai, New York, NY 10028 USA; 6 Institute for Genomics, Biocomputing & Biotechnology, Mississippi State University, Mississippi State, MS 39762 USA

**Keywords:** Agricultural sustainability, Neuroactive compounds, Rumen microbiota, Stress

## Abstract

Gut-brain communication via the peripheral neural network is vital for regulating local digestive function and systemic physiology. Gut microbiota, which produces a wide array of neuroactive compounds, is a critical modulator in this bidirectional dialog. Perturbations in the gut microbiota have been implicated in neurological disorders such as depression and stress. Distinct from humans and other monogastric animals, ruminants possess a unique, microbially dense gastrointestinal compartment, the rumen, that facilitates the digestion of fibrous plant materials. These ruminal microbes are likely key contributors to rumen-brain crosstalk. Unlike certain microbe-derived neuroactive compounds produced in the hindgut that are minimally absorbed and primarily excreted in feces, those generated in rumen can reach the small intestine, where they are largely absorbed and affect central nervous system through systemic regulation in addition to the vagal pathway. Notably, emerging evidence suggests that rumen microbiota dysbiosis under stress is associated with abnormal behavior, altered hormonal and neurotransmitter levels. In this review, we introduce the concept of the rumen-microbiome-brain axis by comparing the anatomical structures and microbial characteristics of the intestine and the rumen, emphasizing the neuroactive potential of rumen microbiome and underlying mechanisms. Advances in this frontier hold tremendous promise to reveal a novel dimension of the gut-microbiome-brain axis, providing transformative opportunities to improve ruminant welfare, productivity, and agricultural sustainability.

## Introduction

The central nervous system (CNS), sensory nervous system (SNS), autonomic nervous system (ANS), and enteric nervous system (ENS) form the neural network that underlies the bidirectional communication between the brain and the peripheral intestine [1]. This gut-brain axis influences key physiologies in the animal host, including neurological, endocrine, and metabolic pathways [2, 3]. The gastrointestinal (GI) tract harbors a vast microbial population that plays a critical role in maintaining homeostasis and linking the brain and gut through unique microbial metabolites and microbe-associated molecular patterns (MAMPs) [4, 5]. Multiple gut microbiota-derived components are neuroactive compounds, including neurotransmitters and their precursors, and neuromodulator that regulate the gut-brain axis and animal behaviors [6, 7]. The interactions between gut microbiota and the brain via the neural network has positioned these microbes as emerging therapeutic targets for treating psychiatric disorders and enhancing mental health in human medicine.

However, ruminants, belonging to herbivorous mammals, possess a specialized digestive segment, particularly the rumen, which is distinct from that of monogastric animals because it is densely filled with microbes that efficiently digest cellulose-rich plants through microbial fermentation. The rumen is also innervated by neurons originating from the SNS, ANS, and ENS, providing the anatomical basis for a fast spatially coded rumen-brain communication similar to the intestines. However, the rumen epithelium contains more layers of stratified squamous cells, lacks typical enteroendocrine cells (EECs), and expresses varied levels of neurotransmitter transporters and receptors compared with intestinal epithelium, indicating a unique rumen-brain communication [8, 9]. Bacterial genera such as *Bifidobacterium* and *Lactobacillus*, known for synthesizing neurotransmitters like gamma-aminobutyric acid (GABA) and acetylcholine, are prevalent in the rumen content [10–12]. Additionally, rumen microbes synthesized short chain fatty acids (SCFAs) are predominantly absorbed through rumen wall, where they can be detected by vagal afferents and enter circulation, ultimately influencing brain function, behavior, and emotion, theoretically in a manner similar to SCFAs in monogastric animals [13, 14]. Notably, ruminants are more heavily reliant on their rumen microbial populations for survival compared to monogastric animals. These more diverse microbial communities due to their ecological specialization [15, 16], likely result in a broader spectrum of rumen microbe-derived neuroactive compounds that facilitate this obligate symbiotic relationship. Moreover, unlike certain microbe-derived neuroactive compounds (e.g., GABA and tryptophan) produced in the hindgut, which exhibit limited systemic availability due to poor absorption, those generated in the rumen can pass into the small intestine, where they are primarily absorbed into circulation and may exert neurophysiological effects [17, 18]. Therefore, the rumen microbiota may exert a more profound or unique influence on the CNS compared to the hindgut microbiota due to the distinct metabolic fate of certain neuroactive compounds.

In the livestock industry, ruminants frequently experience inevitable stressors such as weaning, transportation, and environmental stress, which can suppress appetite and reduce productivity [19, 20]. These stressors are often accompanied by shifts in the rumen microbiota [21, 22]. However, the feedback of these rumen microbial changes on animal stress responses through rumen-brain axis remains uncovered. In this review, we propose the neuroactive potential of the rumen microbiota by drawing parallels to the well-characterized roles of intestinal microbiota and illustrating the anatomical uniqueness of ruminants. We also highlight future directions for translating knowledge of rumen-microbiome-brain crosstalk into practical applications aimed at improving ruminant welfare and promoting sustainability in the livestock industry.

## Gut-brain axis

**Fig. 1:**
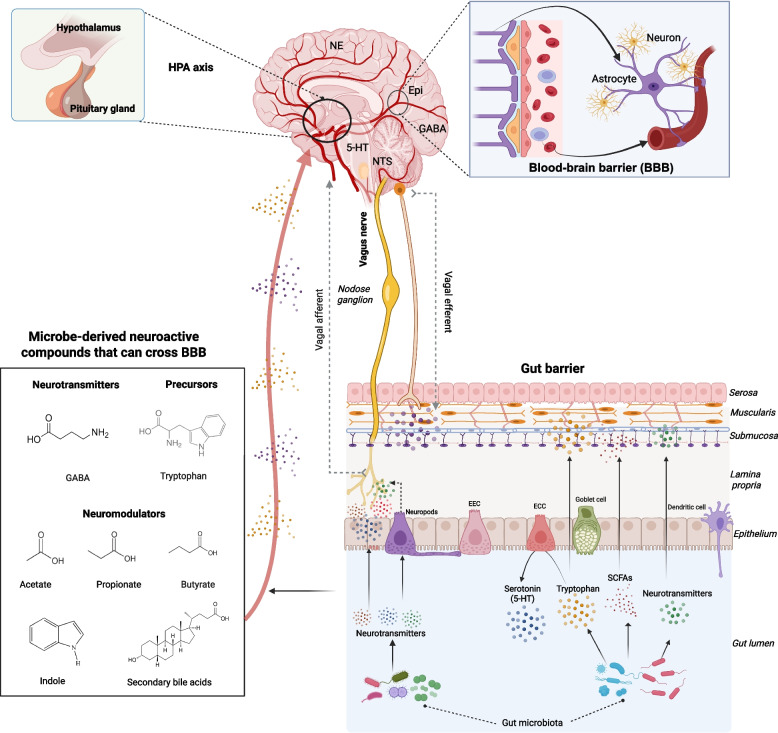
Gut-microbiome-brain axis. The gut-brain axis is composed of several connections between the GI tract and the brain, separated by the gut barrier and the blood–brain barrier (BBB). The BBB, which is made up of brain microvascular endothelial cells in the neurovascular unit, astrocytes, and pericytes, creates a bidirectional network that separates the brain’s vascular system from the systemic blood supply and the gastrointestinal tract’s enteric nervous system. The vagus nerve is shown at the core of this network, with afferent neural (sensory) signals traveling from the gut to the brain and efferent (motor) impulses traveling from the brain to the gut. The vagal afferent system plays a crucial role in the rapid processing and transmission of gut-derived signals. Pseudo-bipolar neurons of the vagal afferent system emerging from the NG, detect peripheral metabolites and neuroactive signals, relaying this information to the NTS in the brainstem to regulate autonomic, endocrine, and behavioral responses. The gut barrier, composed of epithelial cells like EECs, ECCs, neuropods with tight junctions, and mucus from goblet cells, which protects against pathogens. Gut microbe-derived neurotransmitters can activate the enteric neurons and specialized EEC (neuropods), which thereafter activate the afferent vagal fibers and send signals to the CNS. Neurotransmitters that diffuse or are transported across gut epithelial cells can also directly activate vagal afferents and transmit neuroactive signals to the brain. Gut microbe-derived neurotransmitter precursors (e.g., tryptophan) can be converted into neurotransmitters (e.g., serotonin) by host cells and microbes. Gut microbe-derived neuromodulators (e.g., SCFAs) can modulate the neurotransmitter production by enteric neurons and EECs. Additionally, certain gut microbe-derived neuroactive compounds (e.g., GABA, SCFAs, indole, secondary bile acids) can cross the BBB, activate neurotransmitter receptors, affect neurotransmitter production in the brain, and regulate the maturation of astrocytes, thereby affecting CNS function. NE: Norepinephrine; NTS: Nucleus tractus solitarius; NG: Nodose ganglion; EECs: Enteroendocrine cells; ECCs: Enterochromaffin cells; Epi: Epinephrine; HPA: Hypothalamic pituitary adrenal axis; GABA: Gamma-aminobutyric acid; SCFAs: Short chain fatty acids; 5-HT: 5-hydroxytryptamine. This figure was generated using biorender.com

The gut-brain axis is a bidirectional communication network between the GI tract and the CNS (Fig. 1). The CNS receives inputs from the gut in response to mechanical distension, nutrient intake, hormonal factors, and immunological cues. These diverse signals are directly sensed and transmitted by the gut-innervating vagal sensory neurons (afferents) and further integrated with other cues in the nucleus tractus solitarius (NTS) to maintain homeostasis [23, 24]. Moreover, vagal sensory neurons can be activated by gut-derived neurotransmitters like serotonin, which are released from enterochromaffin cells (ECCs) and act on intraganglionic laminar endings that sense intestinal stretch [25, 26]. In addition to activating local vagal sensory neurons, gut signaling molecules can also directly act on the brain via circulation after passing through two primary barriers: the gut barrier and the blood–brain barrier (BBB) [27, 28].

The brain regulates gut functions primarily through neural and hormonal pathways. The brain responds in a timely fashion to sensory signals via the autonomic nervous system, including the sympathetic nervous system and parasympathetic nervous system. Additionally, the GI tract comprises the ENS, the only internal organ having its own independent nervous system, which controls the gut motility, microcirculation, and exocrine and endocrine secretions [29]. While both can operate independently, the ENS receives signals from the brain via autonomic nerves to coordinate actions, such as adjusting motility patterns and modifying the secretion of digestive fluids. Hormonally, the brain influences gut function through the endocrine system by releasing hormones such as corticotropin-releasing hormones, cortisol, and neuropeptides, that regulate appetite, digestion, and motility.

Neuroendocrine coordination within the gut-brain axis comprises complex reciprocal interactions between the nervous and endocrine systems that maintain homeostasis and play critical roles in regulating stress responses. This regulation occurs through hypothalamic control of pituitary hormone secretion, along with the stimulation of neurotransmitters and neuromodulators [30]. The hypothalamus coordinates responses with the pituitary gland, cerebral cortex, brainstem, spinal cord, and sympathetic and parasympathetic preganglionic neurons. These neurons connect to the sympathetic and parasympathetic branches of the ANS, facilitating both afferent signals from the gut lumen to the CNS via enteric, spinal, and vagal pathways, and efferent signals from the CNS to the intestinal wall [31].

## Roles of gut microbiota in the gut-brain axis

Trillions of microbes reside in the GI tract and play vital roles in regulating host digestion, immune function, and behavior [32–34]. These microbes are crucial for brain development [35], neurogenesis [36], and GI tract motility [37]. For example, germ-free mice showed delayed postnatal neurogenesis [38] and exhibited anxiolytic-like behavior compared to conventionally reared specific-pathogen-free (SPF) mice. Reducing the microbiota with antibiotics led to delayed intestinal transit and reduced gut motility [39]. Young germ-free mice that received gut microbiota transplants from old mice exhibited increased hippocampal neurogenesis and intestinal growth [40]. Herein, the gut microbiota influences the nervous system through multiple mechanisms, including the direct synthesis of neurotransmitters that activate the CNS via the vagal pathway or by entering circulation and crossing the BBB, as well as by producing precursors of neurotransmitters and other neuromodulators [41] (Fig. 1).

### Gut microbes synthesize neurotransmitters and other neuroactive compounds

Gut microbes can directly produce neurotransmitters, including glutamate, GABA, serotonin, norepinephrine, dopamine, and acetylcholine, which serve as key chemical messengers regulating neuronal communication. Gut bacteria carry genes encoding glutamine synthetase (*glnA*), glutamate dehydrogenase (*gdhA*), and glutamate synthase (*gltBD*) [42–47], which facilitate the production of glutamate, the principal excitatory neurotransmitter (Table 1). Certain bacteria, such as *Lactobacillus* spp., *E. coli*, *Bifidobacterium* spp., *Parabacteroides*, and *Eubacterium*, can produce the inhibitory neurotransmitter GABA through glutamate decarboxylase, encoded by the *gad* gene [49–51]. *Lactiplantibacillus plantarum*, isolated from the gut of *Drosophila melanogaster*, exhibited the highest acetylcholine levels (2.6 µmol/L) in vitro [59], compared with other bacterial species such as *Bacillus subtilis* (0.05 µmol/L), *Escherichia coli* (0.002 µmol/L), and *Staphylococcus aureus* (0.00039 µmol/L) [64]. Several gut bacteria such as *E. coli*, *Proteus vulgaris*, *Serratia marcescens*, *Bacillus subtilis*, and *Bacillus mycoides* have also demonstrated the ability to synthesize norepinephrine, a neurotransmitter involved in the “fight-or-flight” reaction [55, 56, 61, 63]. Particularly, *Bacillus mycoides* and *Bacillus subtilis* can produce significant amounts of norepinephrine (0.2–2 μmol/L) in their biomass by converting tyrosine into dopamine using tyrosine hydroxylase (*TH*), which is then converted into norepinephrine by dopamine β-monooxygenase [55, 57, 58, 62]. Additionally, gut bacterial strains such as *Staphylococcus pseudintermedius* ED99 and *Staphylococcus aureus* HG003 possess a specific Staphylococcal aromatic amino acid decarboxylase gene (*sadA*-*strip*) responsible for decarboxylating L-3,4-dihydroxyphenylalanine (L-DOPA) and 5-hydroxytryptophan (5-HTP) to mood-related neurotransmitters dopamine and serotonin, respectively [54]. Several gut microbes such as *Akkermensia municipila*, *Bacteroides finegoldii*, and *Alistipes species* release extracellular vesicles (EVs) in the lumen, that are encapsulated with microbial synthesized neurotransmitters including GABA, dopamine and glutamate, and further regulate the CNS [65, 66]. Particularly, *Bacteroides finegoldii* can synthesize high amounts of GABA (4 µmol/L) in its EVs due to enriched enzymes such as GadB involved in the GABA pathway [66].

**Table 1:** Neurotransmitter-producing microbes, associated microbial genes, and their putative regulatory roles in gut-brain axis

Neurotransmitters	Microbial species	Microbial enzymes and genes	Putative regulatory effects
Glutamate	*Lactobacillus plantarum*, *Lactobacillus paracasei*, *Lactococcus lactis*, and *Corynebacterium glutamicum* [45–47] *Escherichia coli* and *Bacillus subtilis* [42, 43]	Glutamine synthetase (*glnA*), Glutamate dehydrogenase (*gdhA*), and glutamate synthase (*gltBD)* [42, 43]	Excitatory neurotransmitter; Transfer intestinal sensory signals to the brain through the afferent fibers of vagus nerve via neuropods [28, 48]
Gamma-aminobutyric acid (GABA)	*Bifidobacterium *spp., *Bacteroides fragilis*, and *Akkermansia muciniphila* [6] *Lactobacillus paracasei*, *Lactobacillus plantarum *[49]	Glutamate decarboxylase (*gad*) [50, 51]	Inhibitory neurotransmitter; Modulate brain-gut microbiome homeostasis by regulating neuronal excitability in brain centers controlling gastrointestinal functions, including motility, secretions, and immune responses; Modulate the GABAergic neurons in the NTS via gut afferents i.e. the vagovagal reflex in the gut-brain microbiome axis [52, 53]
Dopamine	*Staphylococcus *spp. [54] *Bacillus cereus*,* Bacillus mycoides*,* Bacillus subtilis* [55] *Escherichia coli* [56]	Tyrosine hydroxylase (*TH*) [57, 58]	Dopamine precursors like gut-microbe SCFAs modulates dopaminergic activity by altering dopamine transporter binding in brain [41]
Serotonin	*Staphylococcus* spp. [54] *Akkermansia muciniphila* [6]	Staphylococcal aromatic amino acid decarboxylase (*sadA*-*strip*) [54]	Activate the vagal and spinal afferent fibers in gut-brain axis, promote inflammation and act as a trophic factor for neuron and interstitial cell of Cajal development and maintenance [26]
Acetylcholine	*Lactiplantibacillus plantarum* [59]	Not available	Regulate the transmission of excitatory impulses between the enteric neurons in the myenteric plexus [60]
Norepinephrine	*Escherichia coli*, *Proteus vulgaris*, *Serratia marcescens*, *Bacillus subtilis*, and *Bacillus mycoides* [55, 61]	Dopamine β-monooxygenase (*dbh*) [62]	Regulate emotional destress, mood, anxiety-like disorders [63]

Apart from direct neurotransmitter synthesis, gut bacteria can also synthesize neuroactive precursors that serve as substrates for neurotransmitter production and neuromodulators that indirectly regulate nervous system. Over 90% of host serotonin is produced by enterochromaffin cells from tryptophan, a precursor that can be synthesized by bacteria such as *Bacillus subtilis* through tryptophan synthase [67]. Indole, both a precursor of serotonin and a neuromodulator that regulates brain cognition, can be produced by diverse gut bacterial species, such as *E. coli*, *Clostridium sporogenes,* and *Bacteroides* spp. which harbor tryptophanase (*TnaA*) [68]. *Clostridium sporogenes* can also decarboxylate tryptophan to produce tryptamine, a neurochemical molecule affecting the neurological activity of the host [69]. In addition, SCFAs (e.g., acetate, propionate, and butyrate), the major end products of carbohydrate fermentation by gut bacteria, as well as secondary bile acids (e.g., lithocholic acid and deoxycholic acid), synthesized from primary bile acids by gut bacteria such as *Clostridium scindens* and *Clostridium sordellii*, are important neuromodulators that regulate host neurotransmitter synthesis and neuronal development [41, 70, 71].

Moreover, advances in metagenomic sequencing and bioinformatics have enabled the identification or prediction of other gene clusters corresponding to the production or degradation of neuroactive compounds in human gut microbiota [6]. Further studies are needed to confirm their functional roles.

### Regulatory mechanisms of gut microbe-derived neuroactive compounds

Gut microbe-derived neurotransmitters can directly activate receptors on enteric neurons and relay signals to vagal afferents, which are in close proximity to the myenteric plexus of ENS, further affecting the CNS [41, 60]. Neurotransmitters in the gut lumen can also be uptaken by gut epithelial cells by corresponding transporters, and further reach vagal afferent and activate their neurotransmitter receptors expressed on the end terminals of vagal afferent [72]. Gut microbe-derived EVs can cargo microbial synthesized neurotransmitters to the CNS via activation of vagal afferent fibers in the myenteric plexus of ENS [73] or can enter the systemic circulation and cross the blood–brain barrier delivering the protected cargo neurotransmitters directly to the CNS [66]. Gut microbe-derived neurotransmitter precursors such as tryptophan and tyrosine can be converted into serotonin and dopamine by enteric neurons via tryptophan hydroxylase and DOPA decarboxylase [74], and subsequently regulate the CNS through the vagal pathway. Additionally, neuromodulators SCFAs, indole, and secondary bile acids that are uniquely produced by gut microbes can activate specific receptors on enteroendocrine cells, such as free fatty acid receptors (FFARs), Aryl hydrocarbon receptor (AhR), and Farnesoid X receptor (FXR) bile acid receptors, which regulate the expression of gene involved in neurotransmitter, thereby modulating the neurotransmitter synthesis and influencing the activation of ENS and vagal afferents [28, 72]. Moreover, gut epithelium contains specialized neuropod cells [48, 52, 53] that can sense microbial-derived neurotransmitter signals within the lumen and form direct synaptic connections with vagal afferent neurons through axon-like basal processes in the gut mucosa, thereby relaying gut microbial signals to the brain [75]. In addition to transmitting sensory signals, the vagus nerve serves as a physical link in the microbiota-gut-brain axis, presenting a promising therapeutic target for drug delivery via the gut microbiota-vagal pathway [72].

Certain gut microbe-derived neuroactive compounds can be transported to the bloodstream through gut epithelium, across BBB, and influence CNS. Most gut-derived neurotransmitters, such as glutamate, serotonin, dopamine, and acetylcholine, cannot cross the BBB due to their size, polarity, and lack of specific transport mechanisms [76]. However, GABA in the gut lumen can cross the BBB through the GABA transporter 2 (GAT2) system expressed on brain capillary endothelial cells [77, 78] and bind to the specific GABA receptors, such as GABA-A and GABA-B receptors in the pre- and post-GABAergic neuronal terminals, modulating CNS neurotransmitter homeostasis [79, 80]. Microbe-derived tryptophan can cross the BBB via large neutral amino acid transporter 1 and is subsequently converted to serotonin in the brain [67, 72, 81]. SCFAs, indole, and secondary bile acids can also pass through the BBB and regulate brain functions. Acetate improves maturation of astrocytes by serving as an energy source [82] and also enhances the synthesis of neuroactive peptide pro-opiomelanocortin while suppressing neuropeptide Y, further regulating appetite [83]. Propionate and butyrate exhibit neuroprotective properties by attenuating neuropeptide Y depletion [84], with butyrate also modulating brain serotonin production [85]. Additionally, SCFAs enhance BBB integrity by increasing the expression of tight junction proteins, reducing paracellular permeability, thereby regulating the availability of neurotransmitters and their precursor in the brain and further regulate neurodegeneration [86–88] (Fig. 1). In addition, indole derivatives cross the BBB via passive diffusion or by efflux transporters such as P-glycoprotein [89] and can activate AhR signaling in the brain, influencing cognition and neuroprotection [90]. Secondary bile acids have been shown to exert neuroprotective effects in neurodegenerative diseases [91, 92]. They can cross the BBB using the apical sodium-dependent bile acid transporter (ASBT) in enterocytes, modulating the activity of neuronal transporters, such as the dopamine and GABA transporters (DAT, GAT1), thereby enhancing the transport efficiency of these neurotransmitters across neuronal membranes in the brain [93–95].

Taken together, gut microbiota plays important roles in regulating the nervous system, as well as influencing behavior and emotional states. This has led to the concept of psychobiotics, microbes with the capacity to confer mental health benefits [96]. For example, it has been demonstrated that administration of probiotic strains *Bacillus clausii* and *Lactobacillus fermentum* NMCC-14 (10^10^ CFU/mL/d) to mice subjected to acute and subacute restraint stress led to a significant increase in norepinephrine, serotonin, and dopamine levels in the hippocampus and prefrontal cortex [97]. These findings underscore the therapeutic promise of developing gut microbe-derived psychobiotics as interventions to regulate central nervous system function through the gut-brain axis.

## Rumen-microbiome-brain axis in ruminants

### Anatomical basis of the rumen-microbiome-brain axis

In contrast to monogastric animals, ruminants heavily rely on their stomach, particularly the biggest compartment, the rumen and the large microbiome that inhabits it for nutrient digestion. The intricate innervation between the CNS and ENS is essential for the unique motility patterns and circadian rhythms in the rumen [98, 99], especially for coordinating rumination, the unique physiological process of regurgitating, remasticating, and reswallowing feed to optimize fermentation in ruminants. The myenteric plexus, located between the inner circular and outer longitudinal muscle layers of the rumen, comprises ganglia, neuronal soma, and connecting nerve fiber strands (NFS) [98, 100]. The submucosal plexus includes an inner layer, containing ganglia connected by major NFS, and an external layer, innervating the submucosal and mucosal layers in the ruminal wall [98]. In addition to the ruminal wall, the ruminal pillar, a muscular division in the rumen that separates the stomach into different sacs, contains a denser network of primary NFS, arranged in parallel at regular intervals. Anatomical and quantitative distribution of vagus nerve fibers across the rumen was reported in sheep several decades ago [101]. Together, the rumen possesses its own enteric nervous system, which is extensively integrated with vagal innervation to coordinate its complex motor functions, such as rumination.

**Fig. 2:**
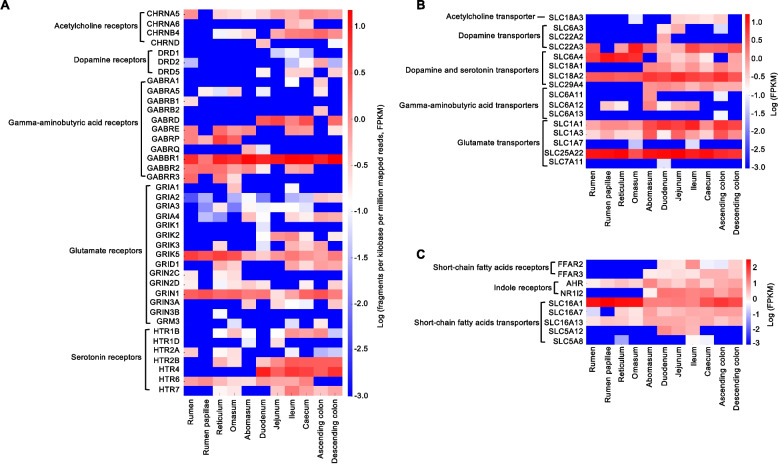
Transcription levels of receptors and transporters of neuroactive compounds along different segments of the gastrointestinal (GI) tract of Hereford cow. The transcription levels of neurotransmitter receptors (**A**) and transporters (**B**), short-chain fatty acids and indoles receptors and transporters (**C**) were analysed in 11 GI segments (rumen, rumen papillae, reticulum, omasum, abomasum, duodenum, jejunum, ileum, caecum, ascending colon, and descending colon) using the publicly available dataset (GSE128075). FPKM: Fragments Per Kilobase of transcript per Million mapped reads

In contrast to the columnar intestinal epithelium, the rumen epithelium is covered with ruminal papillae composed of stratified squamous cells and lacks EECs, such as ECCs and neuropods, which are major sources of neuroactive hormones such as serotonin in the intestine [102, 103], suggesting that host-derived neurotransmitters are less abundant in the rumen. Though neuroendocrine cells were detected in rumen epithelia of sheep and goats using high-resolution single-cell sequencing, the numbers of these cells are much smaller compared with other cell types [104]. However, according to a recent study [105] that conducted a transcriptomic analysis of 91 bovine tissues, including various GI segments (e.g., rumen, rumen papillae, reticulum, omasum, abomasum, duodenum, jejunum, ileum, caecum, ascending colon, and descending colon), the transcription levels of many neurotransmitter receptors and transporters in rumen were comparable to intestine (Fig. 2). For example, the transcription levels of the gamma-aminobutyric acid type A receptor subunit Pi (GABRP), gamma-aminobutyric acid type A receptor subunit Rho3 (GABRR3), glutamate ionotropic receptor kainate type subunit 5 (GRIK5), glutamate ionotropic receptor NMDA type subunit 2 C (GRIN2C), and dopamine and serotonin transporter solute carrier family 6 member 4 (SLC6A4) were higher in the forestomach (rumen, reticulum, and omasum) compared to the true stomach (abomasum) and the intestine. Conversely, other neurotransmitter receptors and transporters, such as the cholinergic receptor nicotinic beta 4 subunit (CHRNB4), dopamine receptors, gamma-aminobutyric acid type A receptor subunit delta (GABRD), glutamate ionotropic receptor AMPA type subunit 4 (GRIA4), glutamate ionotropic receptor kainate type subunit 2 (GRIK2), glutamate ionotropic receptor NMDA type subunit 3 A (GRIN3A), 5-hydroxytryptamine receptor 2B (HTR2B), 5-hydroxytryptamine receptor 4 (HTR4), dopamine and serotonin transporter solute carrier family 18 member A1 (SLC18A1), solute carrier family 29 member 4 (SLC29A4), and glutamate receptor solute carrier family 1 member 1 (SLC1A1) were more abundant in the intestine than in the stomach. Furthermore, we examined the transcription levels of receptors and transporters for microbial metabolites, which play key roles in the gut-brain axis [105]. Notably, the rumen and rumen papillae showed increased transcription levels of the SCFA transporter solute carrier family 16 member 1 (SLC16A1), but significantly lower expression of SCFA receptors and the indole receptor nuclear receptor subfamily 1 group I member 2 (NR1l2) compared with the intestine. However, the other indole receptor, AhR, exhibited higher transcript abundance than NR1l2 in the rumen. Further studies are needed to explore the distribution of these genes among different cell types (e.g., epithelial cells vs. neurons) along the GI tract and the biological differences in transporting and signaling of neuroactive compounds between the rumen and the intestine.

The similarities in the distribution of ENS and vagal afferents in the rumen, compared with those in the intestine, suggest that ruminal afferents are capable of sensing luminal signals. However, with more layers of ruminal epithelial cells, neurotransmitters and other neuroactive compounds produced in the ruminal lumen must diffuse across several epithelial layers before reaching the underlying afferents, which may reduce the efficiency of direct signaling to enteric neurons and vagal afferents. Furthermore, the relatively low abundance of enteroendocrine cells in the rumen may limit the production of host-derived neuroactive hormones and constrain enteroendocrine-CNS signaling pathways. Nevertheless, the unique physiology of rumination, the higher expression of transporters and receptors for certain neurotransmitters and microbe-derived neuroactive compounds in rumen tissue, along with its dense and metabolically active microbiota collectively suggest the existence of a distinct rumen-microbiome-brain axis.

### Neuroactive potential of ruminal microbiota

The rumen hosts a vast and diverse microbial community, dominated by bacteria but with comparatively higher abundances of fungi and protozoa than the intestinal microbiota [106]. While these microbes are essential for fermentation, their potential neuroactive roles remain largely unclear.

**Fig. 3:**
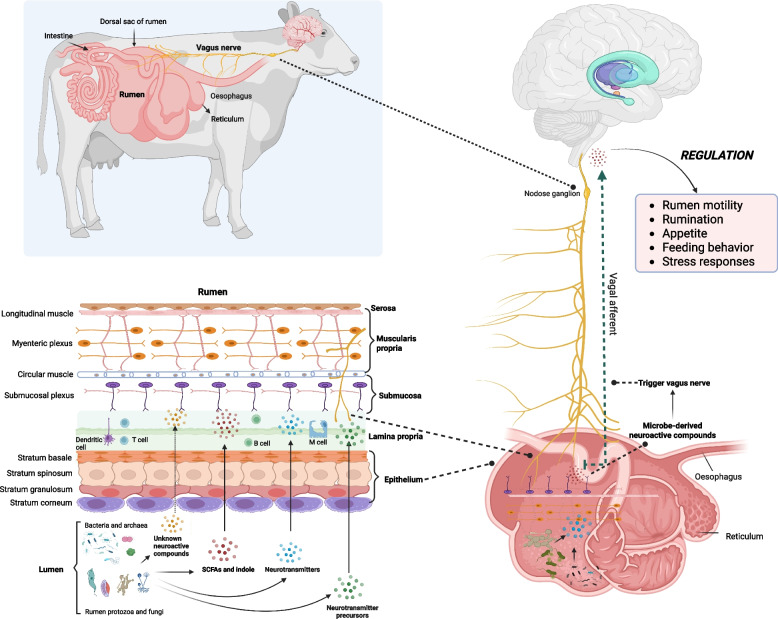
Rumen-microbiome-brain axis. The rumen epithelium is structured with multiple layers of stratified squamous epithelial cells covered with papillae, which provide a robust barrier against the harsh ruminal environment. Unlike the intestine, rumen contains significantly fewer enteroendocrine cells, indicating low levels of host-derived neuroactive hormones (e.g., serotonin). However, the rumen epithelium harbors an intricate ENS network and vagal pathways, facilitating the regulation of digestive processes, feeding behavior, and physiological responses. The rumen microbiota, dominated by bacteria but richer in fungi and protozoa than the intestinal microbiota, also possesses neuroactive potential. Rumen bacteria synthesize neurotransmitters such as glutamate, GABA, serotonin, and norepinephrine, as well as neurotransmitter precursors (e.g., tryptophan) and neuroactive metabolites such as SCFAs and indole. Several protozoa species detected in the rumen also synthesize neurotransmitters, including GABA and dopamine. These rumen microbe-derived neuroactive compounds can diffuse or be transported across the stratified squamous epithelium to activate enteric neurons, stimulate the afferent fibers, and transmit signals to the CNS. Some compounds pass into the intestine, where they may contribute to the gut-brain axis. SCFAs, Short chain fatty acids. This figure was generated using biorender.com

Similar to the gut microbiota, the rumen microbiota consists of bacteria capable of producing neuroactive compounds (Fig. 3). Glutamate in rumen fluid is primarily synthesized by rumen microbes and serves as a central amino acid in nitrogen metabolism, particularly in transamination reactions, and also an energy source for the rumen epithelium [107, 108]. Notably, recent research comparing untargeted metabolomes of ruminal fluid and feces in Nelore steers showed significantly higher concentrations of glutamate (1,111.30 ± 275.75 µmol/L) in the rumen fluid as compared to feces [109], indicating variation in neuroactive compounds along the GI tract of ruminants, particularly between rumen and intestine. As glutamate can be converted to GABA through decarboxylation reaction [110, 111] and rumen microbiota consist of bacteria that encode *gad* gene, rumen fluid likely contains high levels of microbe-derived GABA. Though few studies investigated the neurotransmitter role of glutamate and GABA in the rumen, previous studies found that ruminal glutamate acts as an excitatory neurotransmitter, enhancing the amplitude and frequency of muscular contractions in the longitudinal nerve cords of rumen flatworms such as *Gastrothylax crumenifer* and *Paramphistomum cervi*, while ruminal GABA functions as an inhibitory neurotransmitter, suppressing muscle activity in the ventral nerve cord of these flatworms [112, 113]. Moreover, several rumen microbial species, such as *Butyrivibrio fibrosolvens*, *Selenomonas ruminantium*, and *Treponema bryantii* have shown significant positive association with levels of neurotransmitters like serotonin and norepinephrine in the rumen epithelium [114], indicating their potential role in regulating neurotransmitter synthesis. In addition, rumen-derived EVs have shown to upregulate the expression of genes involved in neuroactive ligand-receptor interactions in *Caenorhabditis elegans*, suggesting their potential role in regulating neurodevelopment [115, 116]. Further, several rumen bacteria such as *Prevotella ruminocola* and *Fibrobacter succinogenes* have demonstrated the capacity to synthesize EVs within the rumen [117]. Nevertheless, the proportion of rumen-derived EVs originating from microbial sources and their potential contribution as neurotransmitter cargo via rumen-vagal pathways or BBB route to CNS remains to be elucidated. Moreover, although few studies reported the capacity of dominant rumen bacteria such as *Prevotella*, *Butyrivibrio*, and *Ruminococcus* [118] to synthesize neurotransmitters, they produce massive quantities of SCFAs through fiber digestion, which act as important neuromodulators [119] that may regulate host-derived neurotransmitters production and modulate the ENS and CNS in ruminants [83, 120].

In addition to bacteria, rumen also hosts up to 50% biomass of protozoa, predominantly represented by genera like *Entodinium, Isotricha, Diplodinium, Epidinium*, and* Dasytricha* [121–123]. These ciliated protozoa directly produce neuromodulator SCFAs through fermentation of fiber and starch [122, 124] and also indirectly regulate the ruminal SCFAs concentration through engulfing the rumen fiber-digesting bacteria and interacting with methanogens that shift the rumen fermentation capability [125]. Though it is largely unknown whether these commensal rumen protozoa can synthesize other neuroactive compounds and further regulate the nervous system of ruminants, several protozoan species detected in the rumen have demonstrated neuroactive potential. For example, *Entamoeba invadens* and *Entamoeba histolytica*, detected in the rumen epithelium of calves [126], have been reported to produce glutamate and GABA in vitro [127]. *Toxoplasma gondii* and *Dictyostelium discoideum*, which account for 5%–7% of classified protozoa species in the rumen fluid of yak and cattle [128], express glutamate decarboxylase isoforms (GadA, GadB) responsible for GABA production [129] and a tyrosine hydroxylase-like enzyme involved in dopamine biosynthesis [130], respectively. However, these protozoa are not classical resident rumen protozoa and may act as transient passengers in the rumen [131]. Further studies are required to determine whether rumen commensal protozoa share similar capabilities and contribute to host neurophysiology.

Furthermore, although the majority of SCFAs are absorbed across the rumen epithelium [132], nutrients that are not fully degraded in the rumen or absorbed therein pass into the intestine, where the rumen microbiota can indirectly influence the ENS and CNS through their contribution to the gut-brain axis. In particular, the small intestine remains the primary site of nutrient absorption, giving certain rumen-derived microbial neuroactive compounds a distinct advantage over those produced in the hindgut, which cannot return to the foregut for absorption and are largely excreted in feces. Although neuroactive compounds generated by the hindgut microbiota can activate enteric and dorsal root nerve signaling, their systemic impact may be limited if absorption in the hindgut is insufficient [133, 134]. For example, rumen-derived microbial GABA and tryptophan, which can cross the BBB, are efficiently absorbed in the small intestine, whereas those synthesized in the hindgut are transported inefficiently across the hindgut epithelium and therefore have limited access to the circulation and CNS [135, 136].

Advances in techniques continue to push this field forward. Untargeted metabolomic analysis discovered additional neuroactive compounds in rumen fluid, such as N-arachidonyl dopamine, an endogenous agonist of Vanilloid Receptor 1 involved in pain sensation and a neuroprotectant [137], and microbe-derived indole derivatives such as 3-indoxyl sulphate, methyl indole-3-acetate, involved in the serotonergic synapse [138]. Further research integrating genomics and metabolomics is necessary to uncover the neuroactive compounds uniquely synthesized by rumen microbes, corresponding microbial genes involved in their biosynthesis, and the regulatory effects on the receptors of the host.

### The relationships between rumen microbiota and stress responses

In the livestock industry, ruminants are routinely exposed to multiple stressors, including separation from the dam and changes in diet during the weaning period [139], loading, crowding, and longer transport duration during the transportation as well as exposure to a new environment, animals after transportation [140], and environmental temperature extremes [19, 141]. During stress, ruminants exhibit a range of behavioral changes, including avoidance of stressors, stereotypic behaviors (e.g., tongue rolling, sham-chewing), reduced feed intake, and altered social responses such as aggression or anxiety [142, 143]. At the neuroendocrine level, stress disrupts neurotransmitter balance, which contributes to decreased health, reproduction, and productivity [144, 145]. In parallel, advances in sequencing technologies have revealed that stress also alters the rumen microbial ecosystem (Table 2).

**Table 2:** Effects of stressors on rumen microbiota, fermentation parameters, host metabolism, and immunity in ruminants

**Type of stress**	**Species or breed of ruminants**	**Microbiome change**		**Change in fermentation Parameters**	**Change in blood parameters**	**Reference**
		**Taxa with increased abundance**	**Taxa with decreased abundance**			
Transportation stress	Simmental Crossbred Cattle (SC), Native Yellow Cattle (NY), and Cattle Yak (CY)	Immediate change after transportation: Phylum: Firmicutes (SC); Genus: Christensenellaceae R-7 (NY and SC), *Lactobacillus* (NY)	Immediate change after transportation: Phylum: Bacteroidetes (NY); Genus: *Prevotella 1* (all breeds), *Butyrivibrio 2* (NY and CY)	Immediate change after transportation: Reduced rumen pH (SC and CY); Increased rumen LPS (SC and CY); Increased propionic acids concentration (all breeds); Reduced rumen lactic acid concentration (SC)	Immediate after transportation: Hormones: Increased cortisol and ACTH (all breeds); Increased serum LPS (CY)	[146]
	Chinese Simmental cross-bred cattle	Phylum: Actinobacteria (Day 30); Genus: *Clostridium* (Day 16), unclassified Porphyromonadaceae (Day 16)	Genus: Unclassified Prevotellaceae (Day 1 or 4 after transport); Species: *Prevotella *sp. tf2-5, Prevotellaceae bacterium*, Lactobacillus brevis*, *Bacteroides *sp. OF04-15BH, *Paraprevotella clara*, *Butyrivibrio *sp. INlla21, *Paraprevotella xylaniphila*	Reduced rumen pH and increased MCP at day 16 post-transport; Increased NH_3_-N from day 4 to day 30 post-transport; Increased acetate, propionate at day 16 then decreased at day 30 post-transport; Increased butyrate at day 30; Increased L-glutamate (Day 1 or 4 after transport)	Hormones: Increased ACTH and cortisol at day 16 and decreased at day 30 after transport; Antioxidant indexes: Reduced T-AOC, SOD, and GSH-PX at day 16 and increased at day 30; Increased MDA at day 16 and reduced at day 30	[147]
	Xianan beef cattle	Species: *Fibrobacter succinogenes*,* Ruminococcus flavefaciens*, *Ruminococcus amylophilus, Prevotella albensis*	Species: *Succinivibrio dextrinosolvens, Prevotella bryantii, Prevotella ruminicola, Anaerovibrio lipolytica*	Reduced rumen pH; Increased acetate and propionate after 7 days of transport	Increased cortisol, ACTH on day 1 after transport	[22]
Weaning stress	Holstein dairy calves	Phylum: Proteobacteria, Firmicutes; Genus: *Pseudoramibacter*,* Butyrivibrio*, *Shuttleworthia*, *Acidaminococcus*, *Dialister*, *Megasphaera*, *Mitsuokella*, *Bulleidia*, *Desulfovibriom*, *Sharpea*	Phylum: Bacteroidetes, Actinobacteria, Verrucomicrobia; Genus: *Bifidobacterium*,* Butyricimonas*,* Odoribacter*,* Parabacteroides*,* Sphingobacterium*,* Elusimicrobium*,* Fibrobacter*,* Streptococcus*,* Succinivibrio*,* Bacteriodes*,* Clostridium*,* Coprococcus*,* Pseudobutyrivibrio*,* Anaerotruncus*,* Oscillospira*,* Ruminococcus*,* Succiniclasticum*,* Sutterella*,* Campylobacter*,* Ruminobacter*,* Acinetobacter*,* Psychrobacter*	NA	NA	[148]
	Holstein dairy calves	Phylum: Fibrobacteres; Genus: *Shuttleworthia*, *Syntrophococcus*, *Fibrobacter*	Phylum: Melainabacteria, Synergistetes; Genus: *Pyramidobacter*	Reduced rumen pH; Increased MCP, acetate, propionate, and butyrate	NA	[149]
	Angus calves	NA	NA	NA	Hormone: Increased cortisol; Neurotransmitter: Increased glutamate	[150]
Heat stress	Lactating Holstein cattle	Phylum: Bacteriodetes; Genus: *Shuttleworthia*, *Anaeroplama*; *Methanobrevibacter*; *Mycosphaerella*, *Filobasidium*, *Issatchenkia*	Phylum: Firmicutes; Genus: *Ruminococcus*,* Desulfovibrio*; fungal genera *Isotricha, Dasytricha, Piromyces*	NA	NA	[12]
	Lactating Holstein cattle	Genus: *Streptococcus*,* Ruminobacter*,* Treponema*, unclassified Enterobacteriaceae, unclassified Bacteroidaceae	Genus: *Acetobacter*	Reduced ruminal pH; Increased lactate, and decreased acetate	NA	[151]
	Holstein Growing heifers	Phylum: Tenericutes, Fibrobacteres*,* Verrucomicrobia, Planctomycetes, Spirochaetes, Patescibacteria, Euryarchaeota, Bacteroidetes Genus: *Treponema 2*,* Kurthia*,* Bifidobacterium*, Rikenellaceae RC9 gut group,* Papillibacter*,* Candidatus Endomicrobium*,* Oscillospira*,* Aneurinibacillus*,* Sphingobacterium*	Phylum: Proteobacteria, Fusobacteria; Genus: *Bacillus*,* Acinetobacter, Lysinibacillus*,* Stenotrophomonas*,* Psychrobacter*,* Clostridium *sensu stricto* 3*,* Pseudomonas*,* Leuconostoc*	Enriched serotonergic pathway		[138]

*ACTH* Adrenocorticotropic hormone, *AOC* Allene oxide cyclase, *GSH-Px* Glutathione peroxidase, *LPS* Lipopolysaccharide, *MCP* Microbial crude protein, *MDA* Malondialdehyde, *NA* Not available/applicable in this study

Transportation is one of the major stressors for farm animals, affecting their health, performance, and product quality. Acute responses to transportation stress lead to elevated stress hormones, increased proinflammatory cytokines, and altered rumen microbiota immediately after transport [22, 146, 147]. These stress responses can last several weeks and cause chronic issues [22, 147]. Stress-induced alterations of rumen microbial population, such as increased lactic acid-producing bacteria *Lactobacillus* and reduced fiber-digesting bacteria *Prevotella*, result in reduced rumen pH and elevated levels of SCFAs, lipopolysaccharide (LPS), and glutamate [22, 146, 147]. These alterations may influence rumen motility, appetite, and emotion of ruminants through activating ENS, vagal, or humoral pathways. The ruminal bacterium *Saccharopolyspora rectivirgula* has been positively correlated with elevated glutamate concentrations in the rumen of beef cattle 16 days post transportation [147]. This species has also been linked to increased oxidative stress post-transport. However, whether elevated ruminal glutamate during transportation intensifies stress responses via vagal pathways and subsequently contributes to oxidative stress remains to be elucidated.

Weaning stress, caused by a shift in diet and separation from mothers, leads to increased vocalization as animals call out for their dams or social companions [152], reduced time grazing, ruminating, and daily feed intake of the calves [150]. These stressful events lead to reduced rumen pH, altered rumen microbiome (e.g., increased *Butyrivibrio* and reduced starch-degrading bacteria such as *Ruminobacter*), increased SCFA levels, and slowing the growth of the calves [139, 148, 149, 153]. A recent study observed increased glutamate concentration in the plasma of weaned calves compared to non-weaned calves [150]. However, the extent to which changes in the rumen microbiota influence the profiles of ruminal neuroactive compounds has yet to be elucidated.

Heat stress has a considerable impact on the livestock industry, particularly for dairy cattle with high milk production and elevated internal heat loads, an issue which is worsened due to global warming [154]. Heat-stressed ruminants experience discomfort, with increased respiration rates, decreased feed intake, and altered rumination behavior, further adversely affecting dairy cow reproduction and milk yield [155, 156]. Rumen microbial communities are also usually disrupted during heat stress [151, 157], with an increase of lactate-producing bacteria, such as *Streptococcus *spp., and amylolytic bacteria, such as *Ruminobacter *spp., that results in higher ruminal lactate concentrations, reduced acetate level, and lower ruminal pH [151]. Neurotransmitter glutamate supplementation can relieve heat stress conditions by improving daily feed intake and weight gain, and reducing stress hormone corticosterone level in sheep, due to its potential role in enhancing rumen fermentation [158]. However, the potential role of rumen glutamate as an excitatory neurotransmitter to activate ENS and enhance rumen contraction during heat stress, and further improve appetite, is largely unexplored. Heat stress alters serotonin/5-HT metabolism by reducing the circulating serotonin levels in dairy calves [159]. Interestingly, the rumen fluid metabolome showed the serotonergic synapse pathway as the most enriched metabolic pathway in growing Holstein heifers experiencing heat stress compared to a non-heat-stressed group [138]. Moreover, heat-stressed heifers exhibited increased levels of indole derivatives, including 3-indoxyl sulfate and methyl indole-3-acetate [138], suggesting that heat stress reshapes rumen microbial metabolism of tryptophan and serotonin, which may in turn influence the CNS through serotonin vagal pathways, as well as the availability of tryptophan and indole metabolites for brain serotonin synthesis.

Emerging evidence suggests that stress-induced shifts in the rumen microbial population likely contribute to changes in the profiles of neuroactive compounds in both rumen fluid and blood. These neuroactive compounds may provide feedback to the brain via the nervous system, influencing behavior, mood, and other physiological states. A recent study showed that oral supplementation of probiotics, particularly the lactic acid-producing bacteria *Enterococcus faecium*, *Lactobacillus acidophilus*, *Lactobacillus casei*, and *Lactobacillus plantarum*, resulted in overall slower chute exit speed and less frequent vocalizations, suggesting that dietary probiotics can improve cattle performance and benefit cattle handling [160]. Future research should aim to uncover whether these supplemented probiotics colonize the rumen and produce neuroactive compounds that exert behavioral and stress-reducing effects.

## Future directions

Single-cell RNA sequencing provides a high-resolution approach to unravel the complexity of rumen epithelial cell populations and has revealed the presence of neuroendocrine cells and neurons within the rumen tissue [104]. The ruminal tissue exhibits a sophisticated multilayered architecture, and emerging single-cell spatial transcriptomics could offer an even more powerful approach by preserving spatial context and enabling the mapping of gene expression within specific cellular niches [161]. However, since vagal afferent fibers are embedded within the rumen wall, but their soma reside in the nodose ganglion rather than in the rumen itself, transcriptomic data alone may be insufficient. Therefore, complementary protein-based approaches (e.g., immunohistochemistry or receptor-specific labeling) will be necessary to verify the expression of vagal afferent receptors in ruminal tissue. Integrating these transcriptomic and protein-based strategies will provide deeper mechanistic insights into vagal signaling and ultimately enhance our understanding of the rumen-brain axis.

A growing body of research has demonstrated that intestinal microbes interact and communicate with the brain and gastrointestinal tract via neurotransmitters [162, 163]. However, the intricate relationship between rumen microbes and the brain remains poorly understood and requires further exploration. Recent advancements in long-read sequencing technologies and the development of metabolomics pipelines tailored for microbiota offer significant potential to identify novel ruminal microbial genes and metabolites [164, 165]. The integration of metagenomic and metabolomic approaches could provide critical insights into the capability of microbes to produce specific neuroactive compounds in the rumen. Furthermore, monitoring the expression levels of neuroactive compound-producing microbial genes and tracing the vagal or circulatory routes of microbe-derived neuroactive compounds under stress conditions could pave the way for understanding how the rumen microbiome responds to stressors. Future research should also examine how stress and rumen microbiota modulate the expression of host transporters and receptors for neuroactive compounds. Alterations in serotonin, dopamine, and GABA transporter and receptor expression in ruminal epithelial and neural cells may critically influence the signaling efficiency between the rumen and the CNS. Filling these knowledge gaps will be essential to unraveling how the rumen microbiome and host interface regulate neuroactive signaling under stress.

Uncovering the rumen-brain-microbiome axis holds immense potential to enhance animal welfare and promote agricultural sustainability. Identifying rumen microbial pathways that produce neuroactive compounds could lead to targeted nutritional or microbial interventions to mitigate stress-related disorders and improve resilience in livestock, which will reduce the need for antibiotics and enhance productivity without compromising animal welfare.

## Conclusions

The rumen-microbiome-brain axis presents an emerging frontier in understanding the bidirectional communication between GI tract and CNS in ruminants, bridged by ruminal microbes, and their influence on host digestion, stress responses, and overall physiology. Recent evidence suggests that stressors common in livestock management disrupt the rumen microbiota, potentially altering the neurochemical milieu, impacting animal behavior and physiology via neural and humoral pathways. However, the mechanistic links between rumen microbial shifts and host neurophysiology have not been comprehensively studied. Advancing our understanding of this axis through integrated transcriptomic, metabolomic, and neurobiological studies will be crucial. In particular, identifying microbial genes involved in neuroactive compound biosynthesis and mapping their effects on host neural circuits offers promising avenues for intervention. Targeted manipulation of the rumen microbiota could enable novel strategies to mitigate stress, enhance animal resilience, and improve welfare. Ultimately, exploring the neuroactive potential of the rumen microbiome not only expands our grasp of host-microbe interactions but also supports the development of sustainable and welfare-conscious livestock systems.

## Data Availability

Not applicable.

## Abbreviations

ACTH: Adrenocorticotropic hormone
AhR: Aryl hydrocarbon receptor
ANS: Autonomic nervous system
ASBT: Apical sodium-dependent bile acid transporter
BBB: Blood–brain barrier
CFU: Colony-forming unit
CHRNB4: Cholinergic receptor nicotinic beta 4 subunit
CNS: Central nervous system
DAT: Dopamine transporter
DOPA: Dihydroxyphenylalanine
ECCs: Enterochromaffin cells
EECs: Enteroendocrine cells
ENS: Enteric nervous system
EVs: Extracellular vesicles
FFARs: Free fatty acid receptors
FXR: Farnesoid X receptor
GABA: Gamma-aminobutyric acid
GABRD: Gamma-aminobutyric acid type A receptor subunit delta
GABRP: Gamma-aminobutyric acid type A receptor subunit Pi
GABRR3: Gamma-aminobutyric acid type A receptor subunit Rho3
GAD: Glutamate decarboxylase
GAT: GABA transporter
GdhA: Glutamate dehydrogenase
GI: Gastrointestinal
GlnA: Glutamine synthetase
GltBD: Glutamate synthase
GRIA4: Glutamate ionotropic receptor AMPA type subunit 4
GRIK2: Glutamate ionotropic receptor kainate type subunit 2
GRIK5: Glutamate ionotropic receptor kainate type subunit 5
GRIN2C: Glutamate ionotropic receptor NMDA type subunit 2C
GRIN3A: Glutamate ionotropic receptor NMDA type subunit 3A
HTP: 5-Hydroxytryptophan
HTR2B: 5-Hydroxytryptamine receptor 2B
HTR4: 5-Hydroxytryptamine receptor 4
L-DOPA: L-3,4-dihydroxyphenylalanine
LPS: Lipopolysaccharide
MAMPs: Microbe-associated molecular patterns
NECs: Neuroendocrine cells
NF: Nodose ganglion
NFS: Nerve fiber strands
NR1l2: Nuclear receptor subfamily 1 group I member 2
NTS: Nucleus tractus solitarius
SadA-strip: Staphylococcal aromatic amino acid decarboxylase
SCFAs: Short-chain fatty acids
SLC16A1: Solute carrier family 16 member 1
SLC18A1: Solute carrier family 18 member A1
SLC1A1: Solute carrier family 1 member 1
SLC29A4: Solute carrier family 29 member 4
SLC6A4: Solute carrier family 6 member 4
SMCTs: Sodium-coupled monocarboxylate transporters
SNS: Sensory nervous system
SPF: Specific-pathogen-free
TH: Tyrosine hydroxylase
TnaA: Tryptophanase
